# QTL-mapping in the obese Berlin Fat Mouse identifies additional candidate genes for obesity and fatty liver disease

**DOI:** 10.1038/s41598-022-14316-5

**Published:** 2022-06-21

**Authors:** Manuel Delpero, Danny Arends, Aimée Freiberg, Gudrun A. Brockmann, Deike Hesse

**Affiliations:** Department for Crop and Animal Sciences, Albrecht Daniel Thaer-Institute of Agricultural and Horticultural Sciences Humboldt-Universität Zu Berlin, Unter den Linden 6, 10099 Berlin, Germany

**Keywords:** Genetics, Metabolic disorders

## Abstract

The Berlin Fat Mouse Inbred line (BFMI) is a model for obesity and the metabolic syndrome. This study aimed to identify genetic variants associated with liver weight, liver triglycerides, and body weight using the obese BFMI sub-line BFMI861-S1. BFMI861-S1 mice are insulin resistant and store ectopic fat in the liver. In generation 10, 58 males and 65 females of the advanced intercross line (AIL) BFMI861-S1xB6N were phenotyped under a standard diet over 20 weeks. QTL analysis was performed after genotyping with the *MiniMUGA* Genotyping Array. Whole-genome sequencing and gene expression data of the parental lines was used for the prioritization of positional candidate genes. Three QTLs associated with liver weight, body weight, and subcutaneous adipose tissue (scAT) weight were identified. A highly significant QTL on chromosome (Chr) 1 (157–168 Mb) showed an association with liver weight. A QTL for body weight at 20 weeks was found on Chr 3 (34.1–40 Mb) overlapping with a QTL for scAT weight. In a multiple QTL mapping approach, an additional QTL affecting body weight at 16 weeks was identified on Chr 6 (9.5–26.1 Mb). Considering sequence variants and expression differences, *Sec16b* and *Astn1* were prioritized as top positional candidate genes for the liver weight QTL on Chr 1; *Met* and *Ica1* for the body weight QTL on Chr 6. Interestingly, all top candidate genes have previously been linked with metabolic traits. This study shows once more the power of an advanced intercross line for fine mapping. QTL mapping combined with a detailed prioritization approach allowed us to identify additional and plausible candidate genes linked to metabolic traits in the BFMI861-S1xB6N AIL. By reidentifying known candidate genes in a different crossing population the causal link with specific traits is underlined and additional evidence is given for further investigations.

## Introduction

Obesity and its related pathologies such as insulin resistance, type 2 diabetes, and fatty liver are symptoms of an imbalanced energy homeostasis^1^. A sedentary lifestyle as well as the (over)consumption of easily available energy-dense food contribute to this imbalance^1^. However, genetic constitution sets the stage for the phenotypic characteristics. Genome-wide association studies (GWAS) in humans have revealed more than 300 single-nucleotide polymorphisms (SNPs) associated with obesity-related phenotypes^2^. Nevertheless, identified loci and underlying genes explain only a minor proportion of the estimated heritability^2,3^.

Mice are ideal model organisms for studying genetic effects because the environmental conditions can be tightly controlled. Furthermore, different inbred mouse lines with distinct but well-defined genetic constitution are available, which can be used to improve our understanding of the genetic architecture of complex phenotypes like obesity. Crosses between inbred lines allow the generation of structured populations. These prerequisites make association studies between genetic loci and phenotypes feasible in relatively small populations with high statistical power^4^. In particular, advanced intercross lines (AIL) allow high resolution QTL mapping by increasing recombination between any two loci^4–7^.

The Berlin Fat Mouse with its different inbred lines (BFMI) were generated initially by crossing various mice from different pet shops, subsequent selection for high body weight and high fat mass^8^, and finally repeated inbreeding between pairs of full sibs to generate a mouse model for the investigation of body weight gain and body composition. The different BFMI sub-lines are genetically closely related. They are all obese, but show different features of the metabolic syndrome^9,10^.

Due to their unique genetic background in combination with the distinct obese phenotypes, the BFMI sublines allow the identification of diverse genetic contributors to the metabolic syndrome. In a cross between the obese line BFMI860-12 and C57BL/6N (B6N) as a lean strain, a major QTL for total fat mass was mapped on chromosome (Chr) 3 (*jObes1*)^11^. The locus was further fine-mapped and characterized, which led to the identification of *Bbs7* as causal gene (6), a gene that contributes to the Bardet-Biedl syndrome in humans. In an AIL between BFMI861-S1 and BFMI861-S2, two genetically very similar lines, we identified QTLs and candidate genes responsible for differences in liver weight, liver triglycerides, gonadal adipose tissue weight, and body weight^7^. The BFMI861-S1 line of this cross does not only carry the mutant *jObes1* allele, it also shows the highest liver weight and liver triglyceride (TG) concentration among all BFMI sub-lines^9^. Therefore, the BFMI861-S1 line is an interesting mouse model to study the genetic architecture of hepatic fat deposition in the context of obesity. In this study, we have generated the advanced intercross of BFMI861-S1 and B6N, where B6N is a lean counterpart to BFMI861-S1 to identify additional genes contributing to the specific phenotype of the BFMI861-S1 sub-line, in particular its fatty liver.

## Material and methods

### Mouse population

58 male and 65 female mice of the AIL BFMI861-S1xC57BL/6N (AIL BFMI861-S1xB6N) in generation 10 were genotyped and phenotyped. The AIL population was generated from an F2 population between an obese BFMI861-S1 male and a lean B6N female. Beginning in generation F1, individuals were randomly mated to mice from the same generation using the program RandoMate^12^.

### Animal husbandry

All animal experiments were approved by the German Animal Welfare Authorities (approval no. G0099/16) and reported in accordance to ARRIVE guidelines. All methods were performed in accordance with the relevant guidelines and regulations.

Mice were maintained under conventional conditions and a 12:12 h light–dark cycle (lights on at 06:00) at a temperature of 22 ± 2 °C. Animals had ad libitum access to food and water. Animals were fed a standard rodent diet containing 16.7 MJ/kg of metabolizable energy, 11% from fat, 26% from protein and 53% from carbohydrates (V1534-000, ssniff EF R/M; Ssniff Spezialdiäten GmbH, Soest, Germany).

### Phenotyping

Animals were analyzed between the age of 4 (after weaning at 3 weeks) and 20 weeks with body weight being recorded weekly. To investigate glucose metabolism, an oral glucose tolerance test (GTT) (week 18) and an intraperitoneal insulin tolerance test (ITT) (week 20, 1 U insulin/kg body weight) were performed as previously described^13^. The area under the curve (AUC) for blood glucose for GTT and ITT was calculated. At 20 weeks, the mice were anesthetized with isofluorane after a fasting period of 2 h and sacrificed by decapitation (in the morning until 12 AM). Several tissues including gonadal adipose tissue (gonAT), subcutaneous inguinal adipose tissue (scAT), and liver were dissected, weighed, shock-frozen in liquid nitrogen and stored until further use at − 80 °C. Body length was measured^14^ and the body mass index (BMI) was calculated using the DuBois equation^15^. Liver TG were assessed as previously described^16^. Plasma free fatty acids (FFA), cholesterol, and TG were measured as described in Schulz et al. ^17^. Plasma insulin and skeletal muscle fat % were measured as previously described^18^.

### Genotyping

DNA isolation was done by salt extraction (5 M NaCl with β-mercapthoethanol and proteinase K) and subsequent ethanol precipitation. Genotypes of all 123 mice were generated by Neogen GeneSeek (Lincoln, NE, USA) using the Mini Mouse Universal Genotyping Array (MiniMUGA; Illumina, San Diego, CA, USA). The MiniMUGA array contains probes targeting 10,171 known SNPs (markers)^19^. Markers were removed when all genotypes were missing or when the marker was not segregating. In addition, to prevent spurious associations, we required that at least two of the genotype groups contained 10 observations each. In case one out of three genotype groups contained less than 10 individuals, these were set to N/A, but the marker was kept. After quality control 1,886 high quality markers were available and used for subsequent QTL analysis (Supplementary Fig. S1). Genotypes are available in supplementary File 1.

### QTL mapping

Linear models were used to investigate the influence of subfamily, litter size, and sex on each phenotype. Kinship correction was performed by including subfamily as a covariate when it significantly affected the specific trait. Because litter size and sex affected each phenotype differently, different statistical models were used for mapping each phenotype, which included significant factors as fixed covariates accordingly.

To minimize the influence of population structure, the genomic inflation factor (λ) was computed. If the genomic inflation factor was above 1.05^20^, results were corrected using λ-correction. To account for multiple testing, significance thresholds were corrected using stringent Bonferroni correction. The number of independent SNPs was determined using the simpleM method^21^. The threshold for significance was set using the number of independent SNPs (1,365) as the total number of tests performed. This resulted in a LOD score (as defined by − log10(*p* value)) after λ-correction above 5.1 to be deemed ‘genome-wide highly significant’ and above 4.4 to be ‘genome-wide significant’. QTL regions were defined by a 1.5 LOD drop from the top marker. Region start and end positions are defined by the first marker upstream and downstream, respectively, that have a drop of 1.5 from the LOD score of the top marker^22^.

To discover additional QTLs for body weight, which might be hidden by the known strong effect QTL of the *jObes1* locus, a variation of multiple QTL mapping (MQM) was used^23^. The single QTL model was adjusted to compensate for the known effect of the *jObes1* locus by including the top marker from the Chr 3 region (SNP gUNC5036315) as an additional cofactor into the model:

$${\text{Body}}\;{\text{weight}} = {\text{ sex }} + {\text{mother}} + {\text{ gUNC5}}0{36315 } + {\text{marker genotype}} + {\text{error}}$$

### Gene expression analysis

Gene expression was measured in RNA isolated from liver of BFMI861-S1 male mice (n = 6) at 10 weeks. RNA was extracted as described in Hesse et al.^24^. Gene expression was measured with the Clariom S Assay for mouse (Thermo Fisher Scientific) using the service of ATLAS Biolabs, Berlin, Germany. Gene expression data of male B6N mice (n = 5) measured with the Clariom S Assay for mouse were downloaded from Gene Expression Omnibus^25^. Probe intensities were log_2_ transformed and quantile normalized. To test for expression differences between BFMI861-S1 and B6N mice, two-tailed t-tests were performed. False positives due to multiple testing were minimized using a Benjamini–Hochberg correction. For statistical analysis and for graphical presentation R: A Language and Environment for Statistical Computing^26^ was used. The Mouse Genome Informatics (MGI) database was used to investigate tissue specific expression of top candidate genes^27^.

### Whole genome sequencing

The BFMI861-S1 parental genome was paired-end sequenced using the Illumina HiSeq (Illumina) platform. Obtained DNA reads were trimmed using trimmomatic^28^ after which trimmed reads were aligned to the mouse genome (MM10, GRCm38.p6) using the Burrows–Wheeler Aligner (BWA) software^29^. Subsequently, SAM files were converted to BAM files, sorted, and indexed using Samtools^30^. (Optical) Duplicate reads were removed using Picard tools v2.19.0 after which indel realignment and base recalibration was done using the GATK v4.1.0.0^31^, according to GATK best practices.

All sequence variants in BFMI861-S1 mice were called using BCFtools ^30^. Variants were further annotated using the Ensembl Variant Effect Predictor (VEP)^32^.

### Candidate genes prioritization

Prioritization of candidate genes in each QTL region was performed as described in Delpero et al.^7^. In brief, genes in a QTL region containing sequence variants between the parental lines BFMI861-S1 and B6N were ranked according to the sum of scores for the functional annotation of coding and non-coding variants, gene expression data, and the Kyoto Encyclopedia of Genes and Genomes (KEGG). Coding sequence variants leading to stop gain/stop loss codons and missense mutations located in functional protein domains were awarded a score of 3 points. A missense variant with either a deleterious or a tolerated SIFT (Sorting Intolerant From Tolerant) value obtained a score of 3 or 1, respectively. Non-coding variants were scored based on their location in potential functional sites. If a non-coding variant was located in the promoter or in a splice site, a score of 3 was awarded; if located in untranslated regions (UTRs), enhancers, or CTCF binding sites (involved in 3D structure of chromatin) the score was 1. Genes differentially expressed in the liver were scored with 2; genes annotated in relevant KEGG metabolic pathways with 1.

## Results

### Phenotypic variation and correlation analysis in the AIL

Animals of the AIL population showed high standard deviation for the collected phenotypes which were expected and are needed for QTL analysis. In detail, body weight was on average 40.01 ± 7.27 g at the end of the experiment (week 20). GonAT weight, scAT, and liver weight were on average 1.53 ± 1.0, 0.65 ± 0.32, and 1.92 ± 0.55 g, respectively. The areas under the curve for blood glucose during GTT and ITT were 21,002 ± 12,415 and 7308 ± 3195, respectively. Liver triglycerides and plasma triglycerides were on average 112 ± 68 µg TG/µg protein and 896 ± 506 µg/ml, respectively. Additional plasma parameters such as plasma cholesterol, plasma FFA, and plasma insulin were on average 44 ± 9 mg/dl, 0.23 ± 0.06 mmol/l, and 7 ± 13 ng/mL, respectively. Skeletal muscle fat % also showed high standard deviation in the AIL population (mean = 19 ± 5%) (supplementary Table S1).

In order to assess the relationship among the phenotypes measured in all AIL mice, Spearman correlation was computed between all the collected phenotypes. Most of the phenotypes (scAT weight, gonAT weight, liver weight, body weight, GTT AUC, and ITT AUC, plasma cholesterol, plasma insulin, BMI, body length, and skeletal muscle fat %) were positively correlated among each other (Table 1).

**Table 1:** Spearman correlation coefficients between the collected phenotypes in the AIL population.

jlm	Liver weight	GonAT weight	ScAT weight	Liver TG/Proteins	GTT AUC	ITT AUC	Plasma TG	Plasma FFA	Plasma cholesterol	Plasma insulin	BMI	Body length	SMuscle fat %
Body weight (20 weeks)	**0.74**	**0.64**	**0.81**	**0.34**	**0.78**	**0.84**	**0.34**	0.08	**0.66**	**0.42**	**0.98**	**0.63**	**0.63**
Liver weight		0.21	**0.43**	0.15	**0.61**	**0.66**	**0.56**	0.16	**0.55**	0.2	**0.68**	**0.62**	0.33
GonAT weight			**0.77**	**0.32**	**0.49**	**0.52**	− 0.07	0.09	**0.36**	**0.35**	**0.66**	0.23	**0.74**
ScAT weight				**0.44**	**0.62**	**0.7**	0.12	0.02	**0.51**	**0.48**	**0.81**	**0.4**	**0.81**
Liver TG/Proteins					0.25	0.33	− 0.29	− 0.03	0.26	0.27	**0.35**	0.08	**0.45**
GTT AUC						**0.74**	**0.39**	0.02	**0.49**	**0.39**	**0.79**	**0.37**	**0.56**
ITT AUC							**0.33**	0.03	**0.62**	**0.39**	**0.84**	**0.44**	**0.59**
Plasma TG								0.28	**0.34**	0.25	0.3	**0.38**	0.05
Plasma FFA									0.25	0.01	0.03	0.14	− 0.01
Plasma cholesterol										0.22	**0.65**	**0.45**	**0.39**
Plasma insulin											**0.43**	0.13	**0.48**
BMI												**0.48**	**0.65**
Body length													0.22

Bold indicates significant correlation after multiple testing correction (*p* < 9.10E-04).

gonAT, gonadal adipose tissue; scAT, subcutaneous adipose tissue; TG, triglycerides; ITT, insulin tolerance test; GTT, glucose tolerance test; AUC, area under the curve; FFA, free fatty acids; BMI, body mass index; SMuscle fat %, skeletal muscle fat percentage.

No significant correlation was detected between gonAT weight and liver weight, gonAT weight and plasma TG, and scAT weight and plasma TG. Liver TG showed a significant positive correlation with body weight at 20 weeks (r = 0.34), gonAT weight (r = 0.32), scAT weight (r = 0.44), and skeletal muscle fat % (r = 0.45). In addition, plasma FFA did not show any correlation with the collected phenotypes.

### QTL mapping

For QTL analysis, different statistical models were used for mapping each phenotype (Table 2). The results revealed genome-wide significant loci on three different chromosomes (1, 3, and 6) associated with one or more of the investigated phenotypes (Table 3). Additionally, a suggestive QTL associated with liver TG was found on Chr 8.

**Table 2:** P values for effects of covariates on each phenotype.

	Covariates		
	Sex	Subfamily	Litter size
Body weight (20 weeks)	**0.00001**	**0.00097**	0.17152
GTT AUC	**2.01E−06**	**0.01**	0.05274
ITT AUC	**0.00025**	**0.00022**	0.12422
GonAT weight	**0.00087**	**0.00029**	**0.01736**
ScAT weight	0.55182	**3.35E−06**	0.11907
Liver weight	**1.07E−09**	**0.00077**	0.19891
Liver TG	0.28798	0.51456	0.48629
Plasma cholesterol	**0.0051**	**0.01481**	0.59906
Plasma FFA	**0.00543**	0.20579	0.50047
Plasma TG	**3.24E−10**	0.11351	0.98701
Plasma insulin	0.52115	**0.00252**	0.54256
Body length	**0.00012**	0.10646	0.08461
BMI	**0.00006**	**0.00062**	0.23395
SMuscle fat %	**0.03634**	**0.00003**	**0.03419**

In bold are represented significant covariates that were included in the model for each trait.

gonAT, gonadal adipose tissue; scAT, subcutaneous adipose tissue; Gluc, blood glucose concentration; ITT, insulin tolerance test; GTT, glucose tolerance test; AUC, area under the curve; FFA, free fatty acids; TG, triglycerides; BMI, body mass index; SMuscle fat %, skeletal muscle fat percentage.

**Table 3:** QTLs identified for different phenotypes in the AIL BFMI861-S1xB6N population.

Phenotype	Age [weeks]	N	QTL region				LOD(BH)	% AIL Var	Mean BFMI/BFMI	Mean B6/BFMI	Mean B6/B6
			Chr	Start [bp]	Top [bp]	Stop [bp]					
Body weight [g]	9	123	3	3,40,66,622	3,59,86,311	4,00,43,158	7.45	8.1	34.21	29.08	28.02
	10	123	3	3,40,66,622	3,59,86,311	4,00,43,158	5.69	9.5	36.27	29.98	29.33
	11	123	3	3,40,66,622	3,81,87,507	4,00,43,158	7.24	9.6	38.04	30.75	29.85
	12	123	3	3,40,66,622	3,59,86,311	4,00,43,158	5.92	9.2	39.54	31.68	30.51
	13	123	3	3,40,66,622	3,59,86,311	4,00,43,158	7.08	11.2	40.93	32.57	31.86
	14	123	3	3,40,66,622	3,59,86,311	4,00,43,158	8.89	13	42.46	33.4	32.37
	15	123	3	3,40,66,622	3,81,87,507	4,00,43,158	7.22	10.4	42.93	33.86	33.2
	16	123	3	3,40,66,622	3,59,86,311	4,00,43,158	7.74	12.7	44.34	34.57	33.53
	16	123	6	0	1,39,19,413	1,75,53,096	5.41	8	43.33	45.14	41.47
	17	123	3	3,40,66,622	3,81,87,507	4,00,43,158	4.97	8.6	44.98	36.07	35.15
	18	123	3	3,40,66,622	3,81,87,507	4,00,43,158	5.24	9.1	45.6	36.21	35.81
	19	123	3	3,40,66,622	3,59,86,311	4,00,43,158	5.63	9.1	45.05	36.13	34.78
	20	123	3	3,40,66,622	3,59,86,311	4,00,43,158	5.07	9.8	46.06	37.54	35.91
ScAT weight [g]	20	121	3	3,40,66,622	3,81,87,507	4,00,43,158	5.8	9.1	0.95	0.52	0.48
BMI [kg/m^2^]	20	123	3	3,40,66,622	3,81,87,507	4,00,43,158	4.94	10.4	4.2	3.76	3.67
Liver weight [g]	20	123	1	15,71,32,066	15,86,63,689	16,84,95,457	4.96	5.6	1.81	1.55	-

ScAT, subcutaneous adipose tissue; BMI, body mass index; QTL, quantitative phenotypes locus; Chr, chromosome number; Start, Top, and Stop, position of the start of the QTL confidence interval, position of the SNP with the highest LOD score, and position of the end of the QTL confidence interval in base pairs, respectively. Positions are given according to the Mouse Genome Version MM10, GRCm38.p6. SNP, single-nucleotide polymorphism. The confidence interval gives the 1.5 LOD drop region of the top SNP position. A LOD score above 5.1 was deemed to be ‘genome-wide highly significant’ and above 4.4 was deemed ‘genome-wide significant’. BH, Bonferroni correction; LOD, logarithm (base 10) of odds; Var %, percentage of total variance in AIL explained. The Mean columns show the phenotypic mean adjusted for significant covariates of homozygous BFMI861-S1, heterozygous, and homozygous B6N animals, respectively.

**Figure 1:**
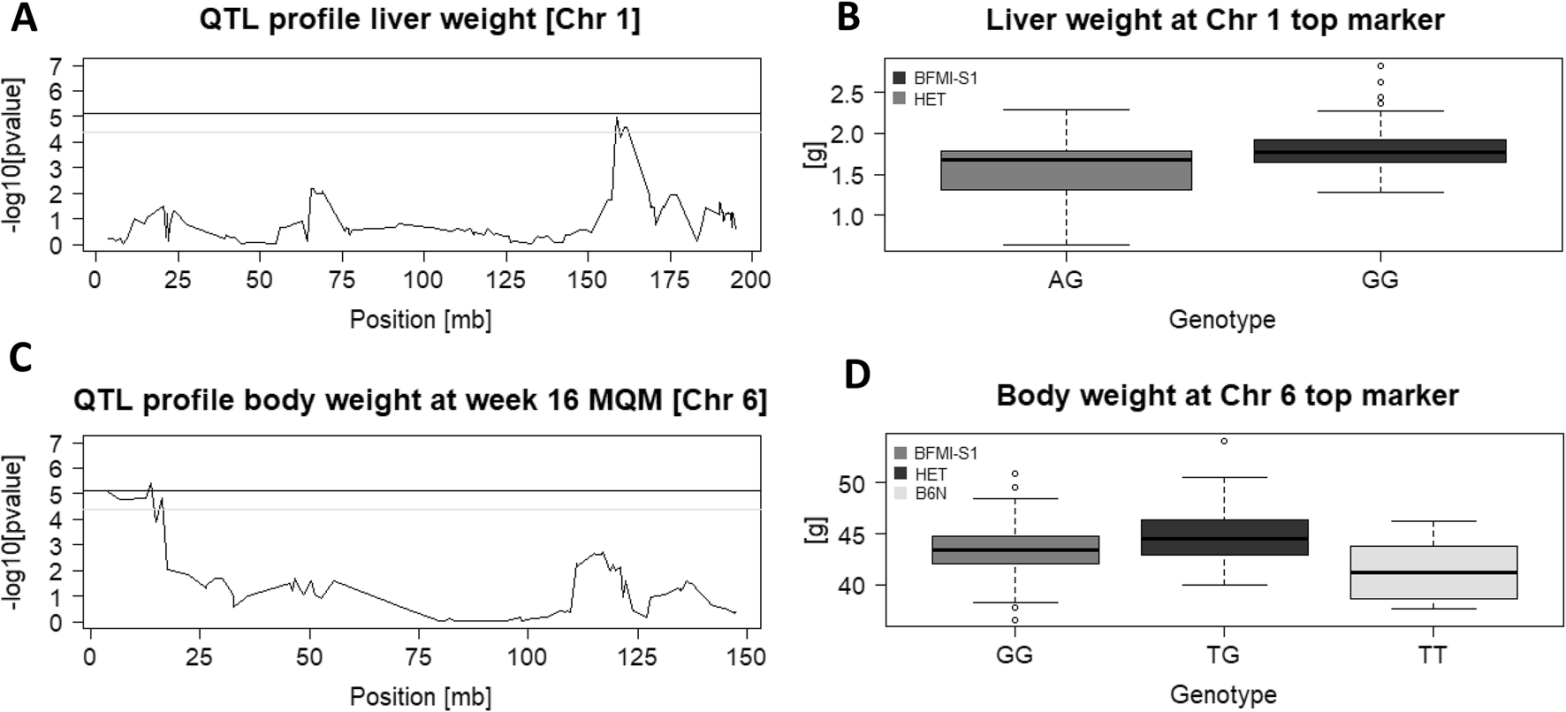
(**A**) QTL mapping curve of the locus on chromosome 1 for liver weight. The black (1%) and grey (5%) horizontal lines mark the significance thresholds; likelihood ratios above the black line are formally highly significant (LOD > 5.1), likelihood ratios above the grey line are formally significant (LOD > 4.4). (**B**) Boxplots for two genotype classes (BFMI-S1, BFMI861-S1 homozygous; HET, heterozygous) at SNP gUNC2036998 which is located at the top position for liver weight. (C) QTL mapping curve on chromosome 6 for body weight at week 16 after performing MQM. (D) Boxplots for all three genotype classes (BFMI-S1, BFMI861-S1 homozygous; HET, heterozygous; B6N, C57BL/6 N homozygous) at SNP gUNC10595065 which is located at the top position for body weight at week 16 after performing MQM.

In detail, the significant QTL on Chr 1 (157,132,066–168,495,457) with a LOD score of 4.96 was associated with liver weight (Fig. 1a). This region contains 89 annotated protein-coding positional candidate genes. The most significant SNP for liver weight in this region was “gUNC2036998” (Chr1:158,663,689). Interestingly, this SNP showed only two genotype classes (homozygous BFMI861-S1 and heterozygous). The liver of homozygous mice carrying the BFMI861-S1 allele was 17% heavier compared to the liver of heterozygous (Het) mice (mean BFMI861-S1 = 1.81 ± 0.25 g, mean Het = 1.55 ± 0.42 g) (Fig. 1b).

**Figure 2:**
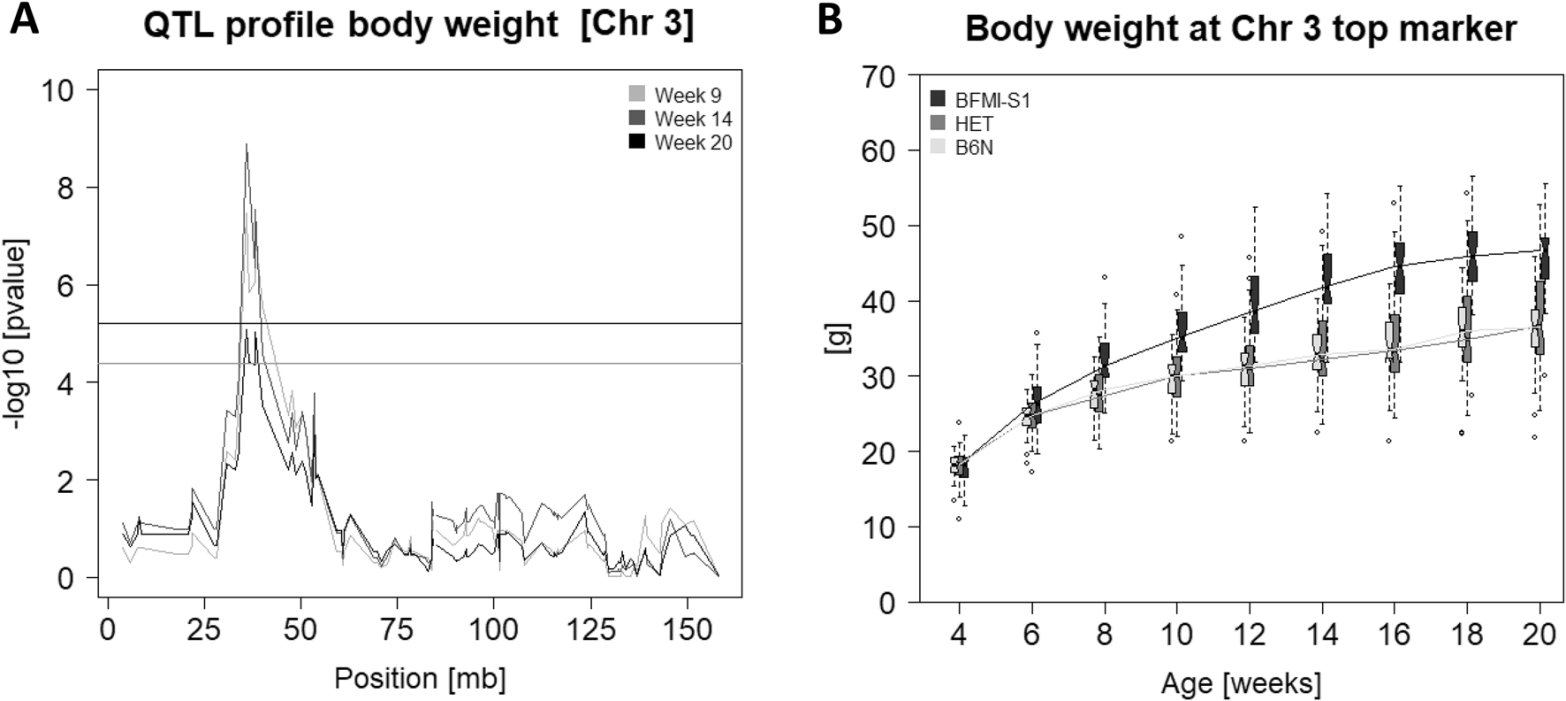
(**A**) QTL mapping curve of the *jObes1* locus on chromosome 3 for total body weight at week 9, 14, and 20. The black (1%) and grey (5%) horizontal lines mark the significance thresholds. (**B**) Boxplots for mice aged 4–20 weeks and curves depicting body weight development. For every time point, boxplots for all three genotype classes (BFMI-S1, BFMI861-S1 homozygous; HET, heterozygous; B6N, C57BL/6 N homozygous) are shown at SNP gUNC5036315, which is located at the top position of the *jObes1* region.

The highly significant region for body weight on Chr 3 (34,066,622–40,043,158) corresponded with the *jObes1* locus that was identified in BFMI mice before^6^. This QTL effect in the AIL BFMI861-S1xB6N persisted at all time points starting from week 9 until week 20 (Fig. 2a). The most significant association (LOD = 8.89) was body weight at week 14 with the top marker gUNC5036315 (Chr3:35,986,311) (Fig. 2b). This marker was 604 kbp away from the *Bbs7* gene that had been identified recently as causal gene for obesity in BFMI mice^6^. At the top marker locus, 14 weeks-old mice homozygous for the BFMI861-S1 allele were 10.09 g heavier than homozygous B6N counterparts. The same region affected also scAT weight (LOD = 5.8), and BMI (LOD = 4.94) with homozygous BFMI861-S1 mice carrying 98% more scAT than B6N homozygous mice (mean BFMI861-S1 = 0.95 ± 0.24 g, mean B6N = 0.48 ± 0.21 g) and 83% compared to heterozygous mice (mean Het = 0.52 ± 0.27 g). In addition, homozygous BFMI861-S1 mice showed 14% BMI increase compared to B6N homozygous mice (mean BFMI861-S1 = 4.20 ± 0.20 kg/m2, mean B6N = 3.67 ± 0.31 kg/m2) and 12% compared to heterozygous mice (mean Het = 3.76 ± 0.33 kg/m2). This region contains 30 annotated protein-coding genes.

When correcting for the top marker of the *jObes1* locus on Chr 3 (gUNC5036315), an additional region associated with body weight at 16 weeks was detected on Chr 6 (0–17,553,096). This region contains 59 protein-coding genes. The most significant SNP of this region was gUNC10595065 (3,919,413; LOD = 5.41) (Fig. 1c). Heterozygous mice showed 9% increase in body weight compared to homozygous B6N mice (mean Het = 45.14 ± 2.91 g, mean B6N = 41.47 ± 3.24 g) and 4.5% increase compared to homozygous BFMI861-S1 mice (mean BFMI861-S1 = 43.33 ± 2.7 g) (Fig. 1d).

A suggestive QTL for liver TG was identified on Chr 8 (86,158,420–106,738,488). Due to the suggestive significance, the region is large containing 179 protein-coding genes. The top marker in this region was “S1H083826428” (Chr8:95,660,710; LOD = 3.93). On average, homozygous mice carrying the BFMI861-S1 allele at this marker showed 90% increase amounts of liver TG compared to homozygous B6N mice (mean BFMI861-S1 = 192 ± 54 µg TG / µg protein, mean B6N = 101 ± 53 µg TG/µg protein) and 83% increase compared to heterozygous mice (mean Het = 105 ± 61 µg TG / µg protein).

### Candidate gene prioritization

Within the confidence intervals of the four QTLs (including the QTL on Chr 8 suggestively associated with liver TG) 357 protein coding positional candidate genes were located. 152 genes were polymorphic between BFMI861-S1 and B6N in protein-coding and/or regulatory regions; 29 on Chr 1, 38 on Chr 3, 22 on Chr 6, and 63 on Chr 8.

In order to identify the most likely candidate genes for each QTL, the 152 polymorphic positional candidate genes were scored according to the decision tree (supplementary Table S2). Tissue expression of top candidate genes was investigated using MGI (27). After applying the prioritization criteria, two genes (*Astn1* and *Sec16b*) located in the region on Chr 1 associated with liver weight ranked with the highest score of 12 and 10, respectively (Table 4). *Astn1* and *Sec16b* carry deleterious missense variants according to the variant effect predictor and are widely expressed including the liver (supplementary Table S3). In addition, both *Sec16b* and *Astn1* show variants in the promoter region and 5-prime and 3-prime UTRs. However, despite variants in regulatory regions *Sec16b* and *Astn1* did not show gene expression differences in the liver.

**Table 4:** Top candidate genes after applying the prioritization criteria.

Gene score	Phenotype	Chr	Positional candidate gene	Type of mutation	P-value liver	FC liver (S1/B6N)
12	Liver weight	1	*Astn1*	Deleterious domain missense, CTCF binds, UTRs, enhancer and promoter variant	0.66493	− 0.03
10		1	*Sec16b*	Deleterious missense, CTCF binds, UTRs, enhancer and promoter variant	0.54953	− 0.01
13	Body weight,	3	*Frem2*	Deleterious domain missense, CTCF binds, UTRs, enhancer and promoter variant	0.02092	0.12
12	scAT weight,	3	*Bbs7*	Tolerated domain missense, CTCF binds, UTRs, enhancer and promoter variant	**0.01346**	− 0.14
12	BMI	3	*Noct*	Tolerated domain missense, CTCF binds, UTRs, and promoter variant	**0.00876**	− 0.17
10	Body weight	6	*Met*	Tolerated domain missense, CTCF binds, UTRs, and enhancer variant	**8.20E**−**06**	− 0.1
9		6	*Ica1*	CTCF binds, UTRs, enhancer and promoter variant	**0.002**	− 0.15
16	Liver TG	8	*Fto*	Tolerated domain missense, stop gained, CTCF binds, UTRs, enhancer and promoter variant	**0.01092**	− 0.09
13		8	*Lpcat2*	Deleterious domain missense, CTCF binds, UTRs, enhancer and promoter variant	0.22981	− 0.05

Bold indicates differentially expressed in liver after Benjamini–Hochberg correction.

scAT, subcutaneous adipose tissue; TG, triglycerides; BMI, body mass index; Chr, chromosome; FC, fold change.

Three genes on Chr 3 (*Frem2*, *Bbs7*, and *Noct*), with a score of 13, 12, and 12, respectively ranked as top candidate genes. Among the candidate genes located in the region on Chr 3 (*jObes1*) associated with body weight from week 9 to 20, *Bbs7*, *Noct*, and *Frem2* all carried missense variants in domains and regulatory region variants and are all widely expressed across tissues including the liver and the nervous system (supplementary Table S3). In addition, *Bbs7* and *Noct* were both downregulated (*p* = 0.01346 and *p* = 0.00876, respectively) in liver of BFMI861-S1 mice compare to B6N, while *Frem2* did not show differences in the expression.

In the region on Chr 6 associated with body weight two genes (*Met* and *Ica1*) with scores of 10 and 9, respectively ranked as top candidate genes. *Met* carries a tolerated missense variant in the IPT (Ig-like, plexins, transcription factors) domain and variants in regulatory regions such as enhancers and untranslated regions. *Ica1* showed variants only in regulatory regions (promoter, CTCF binds, enhancers, and untranslated regions). According to Mouse Genome Informatics both genes are highly expressed in a large variety of tissues (supplementary Table S3). *Met* and *Ica1* were both downregulated in the liver of BFMI861-S1 mice (*p* = 8.2E−06 and 0.00200, respectively).

In the region affecting liver TG on Chr 8, *Fto* and *Lpcat2* were identified as the top candidate genes with scores of 16 and 13, respectively. *Fto* carried a stop gain variant and additional variants in different regulatory regions (promoter, CTCF binds, and enhancer) and was downregulated in the liver of BFMI861-S1 mice (*p* = 0.01092). *Lpcat2* instead carried one deleterious missense variant and regulatory region variants (promoter and enhancer) but did not show expression differences in the liver between BFMI861-S1 and B6N mice. According to the Mouse Genome Informatics database both *Fto* and *Lpcat2* are widely expressed across tissues including the liver (supplementary Table S3).

## Discussion and conclusion

Genome wide association studies (GWAS) on obesity-associated phenotypes identified loci that account in sum only for a small percentage of the total variance of the examined population^2^. Therefore, studies are needed that better allow the identification of genetic effects than most populations do. In order to unravel the genetics behind obesity and hepatic fat deposition, we investigated an AIL population generated from a cross between the obese BFMI861-S1 mouse line and the lean reference strain B6N.

The AIL population used in this study has the advantage of having a high resolution for QTL mapping. Because the examined AIL accumulated recombination over 10 generations, the physical length of the QTL regions is relatively short and as such the number of positional candidate genes is low^4^. In our population, the number of positional candidate genes could be further reduced by removing regions in the genome that are identical between BFMI861-S1 and B6N. Excluding non-polymorphic genes reduced the number of protein coding candidate genes from 357 to 152. In addition, the application of the decision tree led to the prioritization of the most likely candidate genes among the 152 polymorphic genes.

In the region on Chr 1 associated with liver weight, the top candidate genes are *Sec16b* and *Astn1*. *Sec16b* is required for secretory cargo traffic from the endoplasmic reticulum to the Golgi apparatus^33^. Previously, the gene has been linked to increased fat storage in both mice and humans. A human GWAS associated *Sec16b* with differences in body composition^34^. In mice, dysfunctional *Sec16b* was associated with increased body weight^27^. The BFMI861-S1 mice of our study carry a deleterious missense variant of *Sec16b* leading to an impaired protein variant and, in addition, SNPs in the promoter region and 3-prime and 5-prime UTRs. Since *Sec16b* was not differentially expressed between the parental lines BFMI861-S1 and B6N, we hypothesize that the detected deleterious missense variant is responsible for the dysfunction of the encoded protein. Therefore, we consider *Sec16b* as a very strong candidate responsible for the increased liver weight in BFMI861-S1 mice. *Astn1,* the second prioritized candidate gene in the Chr 1 region, is a neuronal adhesion molecule required for the migration of young postmitotic neuroblasts along glial fibers^35^. The gene has not been associated with obesity related phenotypes yet. In BFMI861-S1 mice, *Astn1* carries one deleterious missense variant in the fibronectin type 3 domain. This domain is known to be responsible for interactions with other extracellular matrix (ECM) or cell surface proteins^36^. Therefore, the identified deleterious mutation could reduce the interaction ability of the encoded protein. Additional SNPs between the BFMI861-S1 and B6N in the promoter, 5-prime UTR, enhancers, and CTCF binding sites of *Astn1* could be responsible for the observed downregulation of the gene in the liver of BFMI861-S1 mice versus B6N. This finding confirms that the selected prioritization approach is useful to identify so far unknown candidate genes for the phenotype under investigation which should be considered for follow-up studies.

In the QTL interval for body weight on Chr 3, the prioritization approach identified *Frem2*, *Bbs7*, and *Noct* as the top candidate genes. *Bbs7* has been identify to be the causal gene for elevated fat mass and obesity in all BFMI lines before^6^. Among diverse sequence variants between BFMI and B6N in the *Bbs7* gene, it has been clarified that a large intronic deletion is partially responsible for the high fat content in BFMI mice^37^. This is additional evidence for the prioritization approach being suitable for the correct identification of positional candidate genes in a defined confidence interval.

Only if we accounted for the strong effect of *jObes1* by including the top marker from the Chr 3 region (gUNC5036315) as a cofactor into the model we detected another QTL for body weight on Chr 6. This QTL affecting body weight at 16 weeks partially overlaps with a previously identified QTL for body weight at 10 weeks that was identified in the F2 population BFMI860-12xB6N, which used BFMI860-12 instead of BFMI861-S1 from the current study^11^. In the confidence interval of the QTL identified in our cross, *Met* and *Ica1* are the highest scored top candidate genes. *Met* encodes a receptor tyrosine kinase involved in the transduction of signals from the extracellular matrix to the cytoplasm by binding to hepatocyte growth factor/HGF ligand^38^. In BFMI861-S1 mice *Met* carries a tolerated missense variant in the Ig-like, plexins, transcription factors domain which is involved in the control of cell dissociation, motility, and invasion of extracellular matrices^39^. In addition, *Met* carries SNPs in regulatory regions (enhancers and CTCF binds) which could contribute to the observed downregulation in the liver of BFMI861-S1 mice compare to B6N. According to literature, *Met* is known to be involved in pancreatic-cell death and diabetes^27,40^. Therefore, we consider this gene to be an interesting candidate for the increased body weight of heterozygous mice compared to homozygous BFMI861-S1 and homozygous B6N mice. *Ica1* which is the second prioritized gene of the Chr 6 region encodes for Islet cell autoantigen 1 and plays a role in neurotransmitter secretion^41^. *Ica1* carries several SNPs in the promoter and 5-prime UTR which might cause the downregulation in liver of BFMI861-S1 mice. *Ica1* is also known to be associated with type 1 diabetes mellitus in non-obese diabetic mice^42^ as well as with glucose homeostasis^27^. Therefore, *Ica1* could cause the higher body weight that we observed in heterozygous mice compared to homozygous BFMI861-S1 and homozygous B6N mice.

In the confidence interval of the suggestive liver TG QTL on Chr 8, *Fto* and *Lpcat2* are the most likely candidate genes responsible for hepatic fat accumulation in BFMI861-S1 mice. The same genomic region has previously been associated with liver TG in the Collaborative Cross^43^. In this QTL, we suggested *Fto* to be responsible for the increased amount of TG in the liver of BFMI861-S1 mice. *Fto* has been associated with the body mass index in humans and therefore this gene has been examined extensively^44,45^. *Fto* codes for an RNA demethylase. In mice, both *Fto* knockout and *Fto* missense variants are responsible for fat accumulation and hypertriglyceridemia^46^. The BFMI861-S1 allele carriers of our study carry a stop gain variant at FTO amino acid position 314, which leads to a premature stop codon, and thereby to a shortened protein. Furthermore, BFMI861-S1 mice contain additional missense and promoter variants. The BFMI861-S1 *Fto* haplotype likely contributes as a whole to the observed downregulation in the liver of BFMI861-S1 mice. These findings led us to consider *Fto* as the main contributor to the hepatic fat accumulation in BFMI861-S1 mice. *Lpcat2,* the other prioritized candidate gene is an acetyltransferase^47^. In BFMI861-S1 mice *Lpcat2* carries a missense variant at amino acid position 59 located in the transmembrane helical domain which could affect the function of the protein. In addition, *Lpcat2* carries variants in regulatory regions but is not differentially expressed in liver of BFMI861-S1 mice compare to B6N. According to literature, *Lpcat* proteins are associated with polyunsaturated fatty acid accumulation^48^. Moreover, knockdown of both *Lpcat1* and *Lpcat2* leads to an increase in lipid droplets size^49^. The occurrence of several SNPs in functional regions of *Lpcat2* in BFMI861-S1 mice and its known function in fat accumulation let us to consider this gene as another potential contributor to liver TG accumulation.

In conclusion, our approach identified strong candidate genes that are likely involved in the development of obesity and fatty liver disease in our Berlin Fat Mouse model. However, although we have prioritized candidate genes using the available information, we cannot completely rule out that one of the other polymorphic genes was wrongly discarded, or that non-protein coding regions might be causal. Nevertheless, the natural mutations found in this study in the Berlin Fat Mouse Inbred line BFMI861-S1 contribute to our understanding of which genes impact obesity and hepatic fat storage. These findings help to clarify and support the role of known candidates. The examined mouse model and the applied gene prioritization approach allow to unravel the effects of the identified QTL regions and to link genes with observed phenotypes. Additional studies on the candidate genes should be performed to discover by which molecular mechanisms they contribute to the development of obesity and liver associated diseases in mouse models but also in humans.

## Supplementary Information

Supplementary Information 1.

Supplementary Information 2.

Supplementary Information 3.

Supplementary Information 4.

Supplementary Information 5.

## Data Availability

DNA sequencing data were deposited at the NCBI Sequence Read Archive (SRA) under BioProject ID: PRJNA717237 and is available at: https://www.ncbi.nlm.nih.gov/bioproject/717237. BFMI861-S1 gene expression data measured with Clariom™ S Assay for mouse were deposited at the ArrayExpress Archive (accession E-MTAB-11512).
