# Supplementary figures and images for: QTL-mapping in the obese Berlin Fat Mouse identifies additional candidate genes for obesity and fatty liver disease
Source: Sci Rep. 2022 Jun 21;12:10471. doi: 10.1038/s41598-022-14316-5 (PMC9213485; doi:10.1038/s41598-022-14316-5)

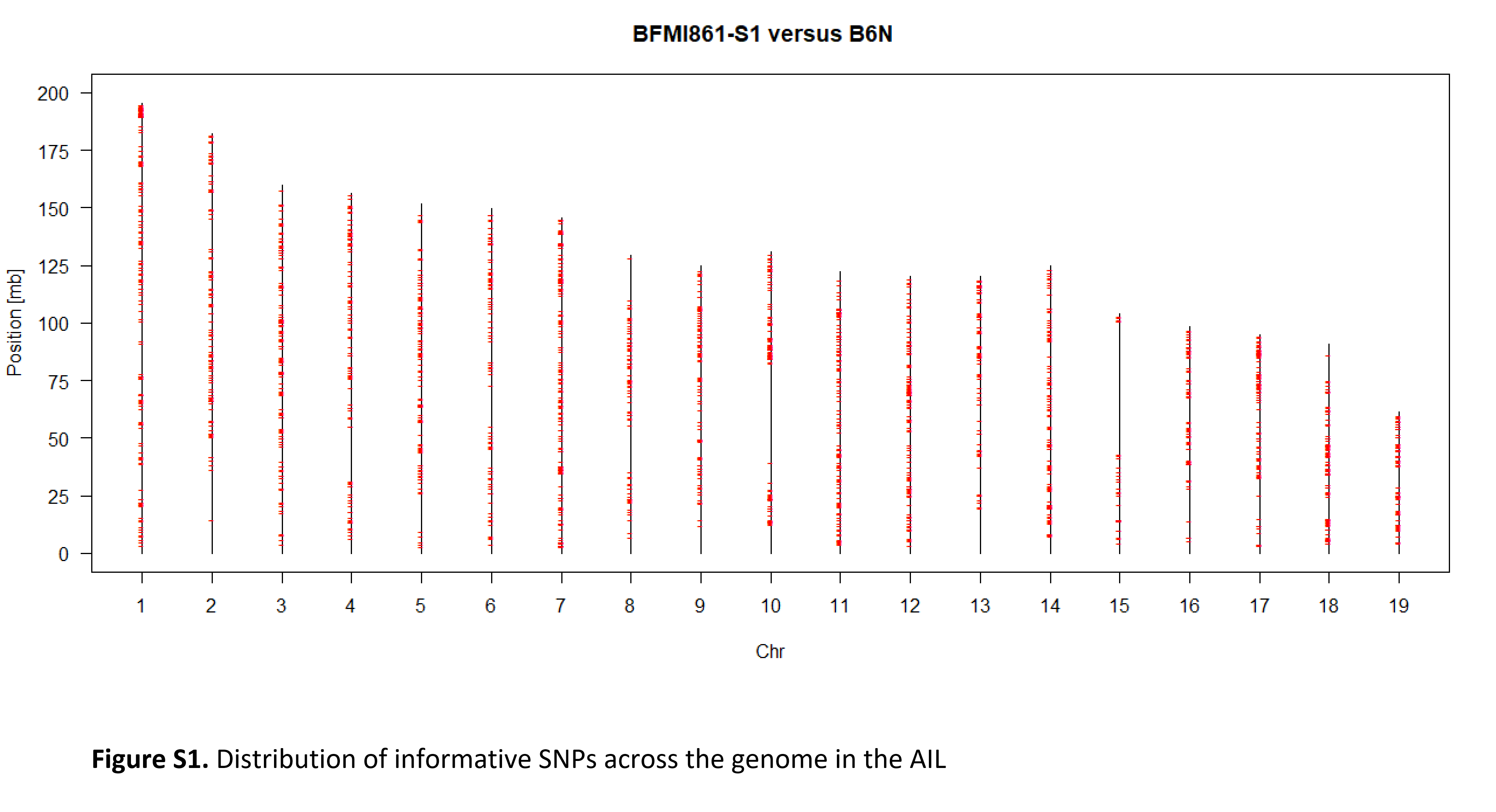

Supplement: Supplementary file 1 — Supplementary Information 1. [file 41598_2022_14316_MOESM1_ESM.png]
